# TCR β chain repertoire characteristic between healthy human CD4+ and CD8+ T cells

**DOI:** 10.1042/BSR20231653

**Published:** 2024-03-06

**Authors:** Ge Li, Yaqiong Chen, Yinji Liu, Zhenfang Gao, Ruiyan Jia, Zhonglin Lv, Yuxiang Li, Zhiding Wang, Gencheng Han

**Affiliations:** Beijing Institute of Basic Medical Sciences, Beijing 100850, China

**Keywords:** CD4, CD8, T cell, TCR Repertoire

## Abstract

T cell is vital in the adaptive immune system, which relays on T-cell receptor (TCR) to recognize and defend against infection and tumors. T cells are mainly divided into well-known CD4+ and CD8+ T cells, which can recognize short peptide antigens presented by major histocompatibility complex (MHC) class II and MHC class I respectively in humoral and cell-mediated immunity. Due to the Human Leukocyte Antigen (HLA) diversity and restriction with peptides complexation, TCRs are quite diverse and complicated. To better elucidate the TCR in humans, the present study shows the difference between the TCR repertoire in CD4+ and CD8+ T cells from 30 healthy donors. The result showed count, clonality, diversity, frequency, and VDJ usage in CD4+ and CD8+ TCR-β repertoire is different, but CDR3 length is not. The Common Clone Cluster result showed that CD4+ and CD8+ TCR repertoires are connected separately between the bodies, which is odd considering the HLA diversity. More knowledge about TCR makes more opportunities for immunotherapy. The TCR repertoire is still a myth for discovery.

## Introduction

When pathogen and antigen are encountered and processed by antigen-presenting cells (macrophage, dendritic cell, B cell, etc.), then short peptides presented through major histocompatibility complex (MHC) molecules to recognize T-cell receptors (TCRs) on the surface of T cells [1]. TCR signaling cooperated with costimulatory molecules, cytokines, integrins, chemokines, and metabolites, which drives T cells to differentiate into CD4+ and CD8+ T cells [2]. CD4+ T cells can differentiate into T helper type 1 (Th1), Th2, Th17, follicular helper T, and regulatory T (Treg) cells. CD8+ effector T cells fight against pathogens at initial exposure, and memory T cells provide defense against future infection [3]. The immune balance was delicately manipulated [4,5].

TCR dynamically changes by the antigens of the immune system faced, such as tumors and infection, which involves HLA diversity and shows the unique TCR repertoire for an individual. The TCR repertoire changes during the time and the antigen, and TCR repertoire analysis is an important way to comprehensively understand the TCR’s nature. TCR signaling impacts the fate of T cells, including expansion, differentiation, and antigen recognition, which is still unclear the contribution of TCR difference.

The emergence of high-throughput sequencing technology and bioinformatics provides opportunities to analyze and annotate immune repertoire data, which can reveal the meaning of immune prediction and progression (tumor microenvironment characterization, minimal residual disease assessment, transplantation, autoimmune disease, and immune checkpoint inhibitor effective evaluation) [6–8] and target antigens (HIV, HBV, HCV, SARS, CoV-2, cancer, etc.) [9,10].

In our previous studies, we focus on MHC-I [11] and MHC-II [12] presentation function in diseases, and TCR repertoire diversity and recognition of MR1 [13]. In the present study, we focus on the TCR repertoire difference between CD4+ and CD8+ T cells. The meaning of the TCR repertoire is still waiting to be found.

## Methods

### Sample

CD4+ and CD8+ T cells’ TCR-β repertoire data of 30 healthy donors were collected with ImmunoSEQ Analyzer 3.0 (Adaptive Biotechnologies) from three different studies (Table 1 and Supplementary Table S1) [14–16]. The alignment of sequencing reads on V, D and J segments of TCR, defined according to IMGT (THE INTERNATIONAL IMMUNOGENETICS INFORMATION SYSTEM, www.imgt.org), assembly of aligned sequences into clonotypes, conversion from nucleotides into amino acid sequences, and computation of the sequencing counts were performed and retrieved from immuneACCESS by Adaptive ImmunoSEQ software.

**Table 1:** Sample Overview

Sample name	Total templates	Productive templates	Fraction productive	Productive rearrangements	Max. productive frequency	Common rearrangements	Percentage	Locus
HC002 CD4	126513	107979	0.8535	95444	0.001102066	3056	3.2018775	TCRB
HC002 CD8	164646	140739	0.8548	72012	0.021308947		4.2437372	TCRB
HC003 CD4	109975	86245	0.7842	71295	0.017160416	1161	1.6284452	TCRB
HC003 CD8	61117	47636	0.7794	20657	0.044504158		5.6203708	TCRB
HC005 CD4	57076	45179	0.7916	36199	0.008388854	801	2.2127683	TCRB
HC005 CD8	124774	93023	0.7455	18431	0.09732002		4.3459389	TCRB
HC006 CD4	133301	111154	0.8339	79930	0.046170179	843	1.0546728	TCRB
HC006 CD8	43443	30638	0.7052	15309	0.10144265		5.5065648	TCRB
HC010 CD4	56214	46320	0.824	35809	0.003044041	968	2.703231	TCRB
HC010 CD8	25337	20675	0.816	14902	0.018186215		6.4957724	TCRB
HC012 CD4	90220	74363	0.8242	64389	0.00285088	1395	2.1665191	TCRB
HC012 CD8	109537	92737	0.8466	31827	0.105125248		4.383071	TCRB
HC013 CD4	96013	77464	0.8068	58254	0.008894454	1295	2.2230233	TCRB
HC013 CD8	138109	106564	0.7716	24571	0.06430877		5.2704408	TCRB
HC016 CD4	140692	117818	0.8374	98658	0.00174846	1579	1.6004784	TCRB
HC016 CD8	64290	52921	0.8232	31364	0.014474405		5.0344344	TCRB
HC017 CD4	121810	99023	0.8129	83534	0.003302263	1187	1.4209783	TCRB
HC017 CD8	73790	60026	0.8135	28235	0.038350049		4.2040021	TCRB
HC036 CD4	196592	161515	0.8216	83807	0.006092313	695	0.8292863	TCRB
HC036 CD8	76456	58523	0.7654	12515	0.043828923		5.553336	TCRB
HC129 CD4	120751	100578	0.8329	67721	0.009435463	429	0.6334815	TCRB
HC129 CD8	59761	50076	0.8379	11234	0.118759483		3.8187645	TCRB
HC159 CD4	80879	63991	0.7912	32040	0.006219625	241	0.7521848	TCRB
HC159 CD8	54094	43665	0.8072	9044	0.073903583		2.6647501	TCRB
HC166 CD4	150333	121735	0.8098	92214	0.020733561	844	0.9152623	TCRB
HC166 CD8	112354	63535	0.5655	18372	0.202754393		4.5939473	TCRB
HC176 CD4	186443	149597	0.8024	70538	0.128398299	280	0.3969492	TCRB
HC176 CD8	84374	60897	0.7218	8104	0.099430189		3.4550839	TCRB
HC186 CD4	560618	406284	0.7247	99401	0.071339257	841	0.8460679	TCRB
HC186 CD8	67833	55642	0.8203	12624	0.246342689		6.6619138	TCRB
HC206 CD4	255093	211660	0.8297	135464	0.002007937	609	0.4495659	TCRB
HC206 CD8	28689	23564	0.8214	11123	0.089373618		5.4751416	TCRB
HC222 CD4	180652	151462	0.8384	95629	7.59E-04	1120	1.1711928	TCRB
HC222 CD8	54119	45084	0.8331	22468	0.038772069		4.9848674	TCRB
HC104 CD4	149197	122466	0.8208	116033	2.71E-04	2191	1.8882559	TCRB
HC104 CD8	72802	59809	0.8215	57912	3.72E-04		3.7833264	TCRB
HC107 CD4	187370	153690	0.8202	95262	0.003713731	1728	1.8139447	TCRB
HC107 CD8	207076	184016	0.8886	35117	0.117768444		4.9206937	TCRB
HC109 CD4	231257	185593	0.8025	166579	5.16E-04	6380	3.8300146	TCRB
HC109 CD8	191794	154732	0.8068	139732	7.21E-04		4.5658833	TCRB
HC110 CD4	134971	102322	0.7581	83680	0.007831992	2770	3.3102294	TCRB
HC110 CD8	165299	131607	0.7962	102114	0.006651776		2.7126545	TCRB
HC113 CD4	231297	183737	0.7944	151538	6.47E-04	5128	3.3839697	TCRB
HC113 CD8	204201	165596	0.8109	146897	0.002181307		3.4908814	TCRB
HC114 CD4	216996	172368	0.7943	151966	6.74E-04	5169	3.4014187	TCRB
HC114 CD8	219316	176355	0.8041	161032	4.08E-04		3.209921	TCRB
HC115 CD4	193074	155612	0.806	121808	0.001200618	4390	3.6040326	TCRB
HC115 CD8	195621	159044	0.813	122239	0.018613486		3.5913252	TCRB
HC116 CD4	217393	173663	0.7988	145613	0.002447208	3890	2.6714648	TCRB
HC116 CD8	167711	133433	0.7956	105826	0.040529493		3.6758453	TCRB
HC117 CD4	204301	166770	0.8163	137085	0.004530581	3883	2.8325491	TCRB
HC117 CD8	195238	160182	0.8204	95553	0.017741513		4.0637133	TCRB
HC118 CD4	108786	81709	0.7511	75788	0.001467944	1136	1.498918	TCRB
HC118 CD8	50994	38331	0.7517	36514	0.002013512		3.1111355	TCRB
HC119 CD4	214870	168472	0.7841	153736	3.38E-04	5885	3.8279908	TCRB
HC119 CD8	199913	156684	0.7838	142975	0.008093297		4.1161042	TCRB
HC120 CD4	114831	87696	0.7637	82457	7.76E-04	1567	1.9003844	TCRB
HC120 CD8	74693	57409	0.7686	54639	9.89E-04		2.8679149	TCRB
HC121 CD4	101637	75795	0.7457	68943	0.006740194	1586	2.3004511	TCRB
HC121 CD8	96616	73740	0.7632	49813	0.084478095		3.1839078	TCRB

### Data analysis

Unless otherwise specified, unique productive TCRβ sequences were defined by CDR3 nucleotide sequence and V and J gene. To identify unique productive TCRβ sequences, individual samples were downloaded from the ImmunoSEQ software and analyzed by VDJtools [17], and productive rearrangements were filtered. VDJtools is a software framework that can analyze TCR repertoire processing tools and allows applying a diverse set of post-analysis strategies. Basic statistics and segment usage module include general statistics (clonotype and read count, number and frequency of non-coding clonotypes, convergent recombination of CDR3 amino acid sequences) [17]. Variable and joining segment usage profiles and their pairing frequency in rearranged receptor junction sequences [17]. Repertoire overlap module includes routines for computing sets of overlapping clonotypes and their characteristic [17]. Diversity analysis includes routines for visualizing clonotype frequency distribution and computing repertoire diversity estimates [17]. Sample clustering is based on computed repertoire similarity measures [17]. When analyzed by amino acid sequence, unique productive TCRβ sequences were defined by CDR3 amino acid sequence. From these, sample template counts across unique productive TCRβ sequences were normalized to the frequency of detection [15].

## Result

### Count, clonality, diversity, and frequency in CD4+ and CD8+ TCR-β repertoire

**Figure 1:**
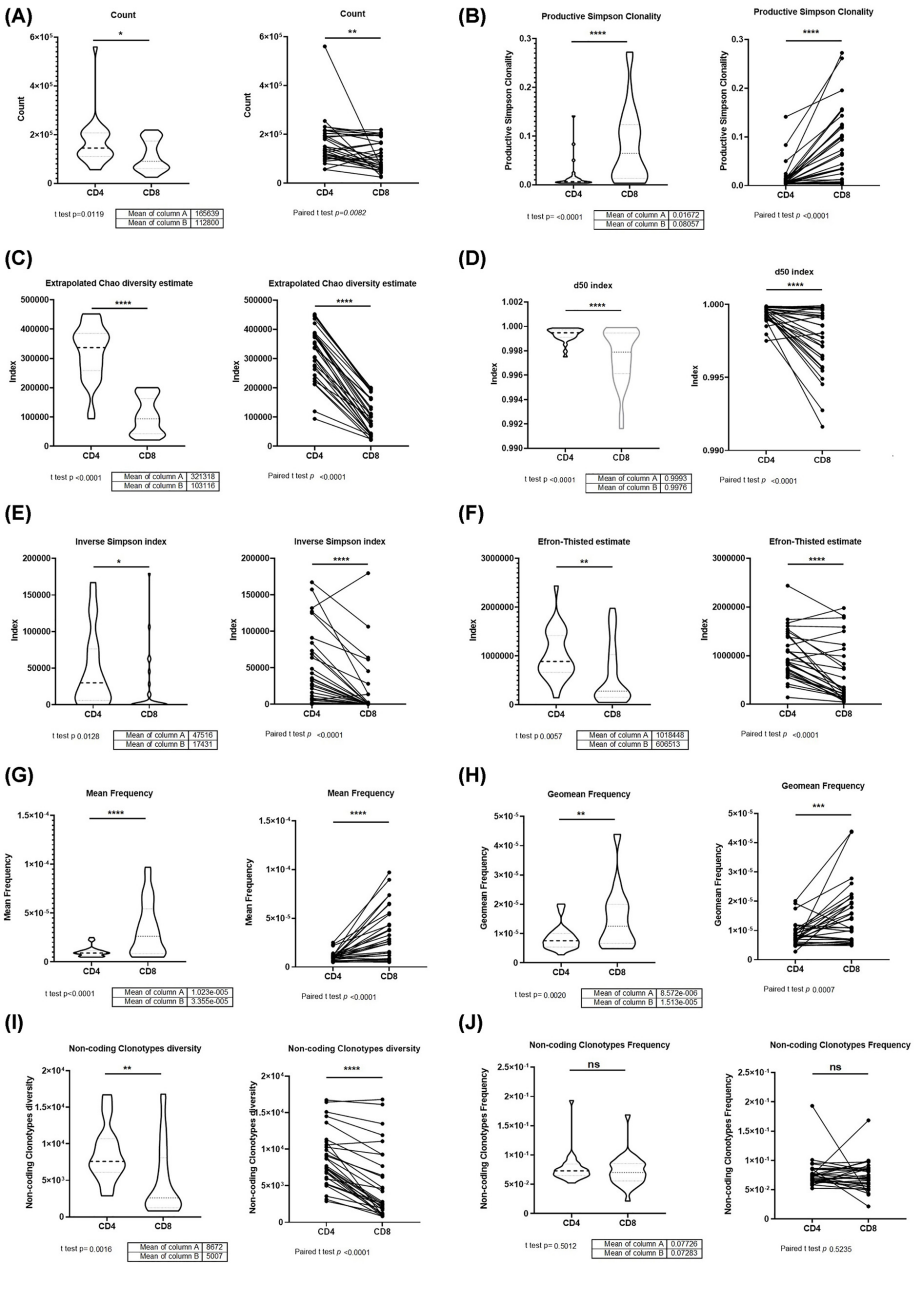
Clonality, count, diversity, and frequency in CD4 and CD8 TCR-β repertoire (**A**) The number of samples reads (Count); (**B**) Productive Simpson Clonality; (**C**) Extrapolated Chao diversity estimate (chaoE); (**D**) d50 index; (**E**) Inverse Simpson Index; (**F**) Efron-Thisted estimate; (**G**) Mean clonotype frequency; (**H**) Geometric mean of clonotype frequency; (**I**) Number of non-coding clonotypes; (**J**) Frequency of reads that belong to non-coding clonotypes (*N* = 30; Left is non-paired *t*-test of CD4 and CD8 TCR-β repertoire as two groups; Right is paired *t-*test of CD4 and CD8 TCR-β repertoire for individual; ns, no significant; *P<0.05; **P<0.01; ***P<0.001; ****P<0.0001).

The counts of CD4+ TCRβ repertoire (Mean 165639) from 30 healthy donors are more than CD8+ TCRβ (Mean 112800), which compared with a group (*P*=0.0119) or individual (*P*=0.0082, Figure 1A). The productive Simpson clonality of CD4+ TCRβ repertoire from healthy donors is less than CD8+ TCRβ compared with group or individual (Figure 1B). The diversity of CD4+ TCRβ repertoire is estimated by Extrapolated Chao diversity estimate, d50, Inverse Simpson index, and Efron-Thisted estimate, which are more than CD8+ TCRβ compared with a group or individual (Figure 1C–F). The mean clonotype frequency of CD4+ TCRβ repertoire from healthy donors is less than CD8+ TCRβ compared with a group or individual (Figure 1G,H). Non-coding clonotypes diversity of CD4+ TCRβ repertoire from healthy donors is less than CD8+ TCRβ compared with a group or individual (Figure 1I), non-coding clonotypes frequency of CD4+ and CD8+TCRβ repertoire from healthy donors are no significant (Figure 1J).

The Productive Simpson Clonality is calculated for a sample as the square root of Simpson’s diversity index for all productive rearrangements. Values near 1 represent samples with predominant rearrangements. Clonality values near 0 represent more polyclonal samples. The estimates computed on original data could be biased by uneven sampling depth (sample size), of those only chaoE is properly normalized to be compared between samples. d50 A method for identifying normal immune status or abnormal immune status in an individual, wherein a normal immune status is characterized by the presence of a greater diversity of clonotypes represented by the significant percentage of the total number of cells, and an abnormal immune status is characterized by the presence of a significantly lower number of clonotypes represented by the significant percentage of the total number of cells [18].

### CDR3 length in CD4 and CD8 TCR-β repertoire

**Figure 2:**
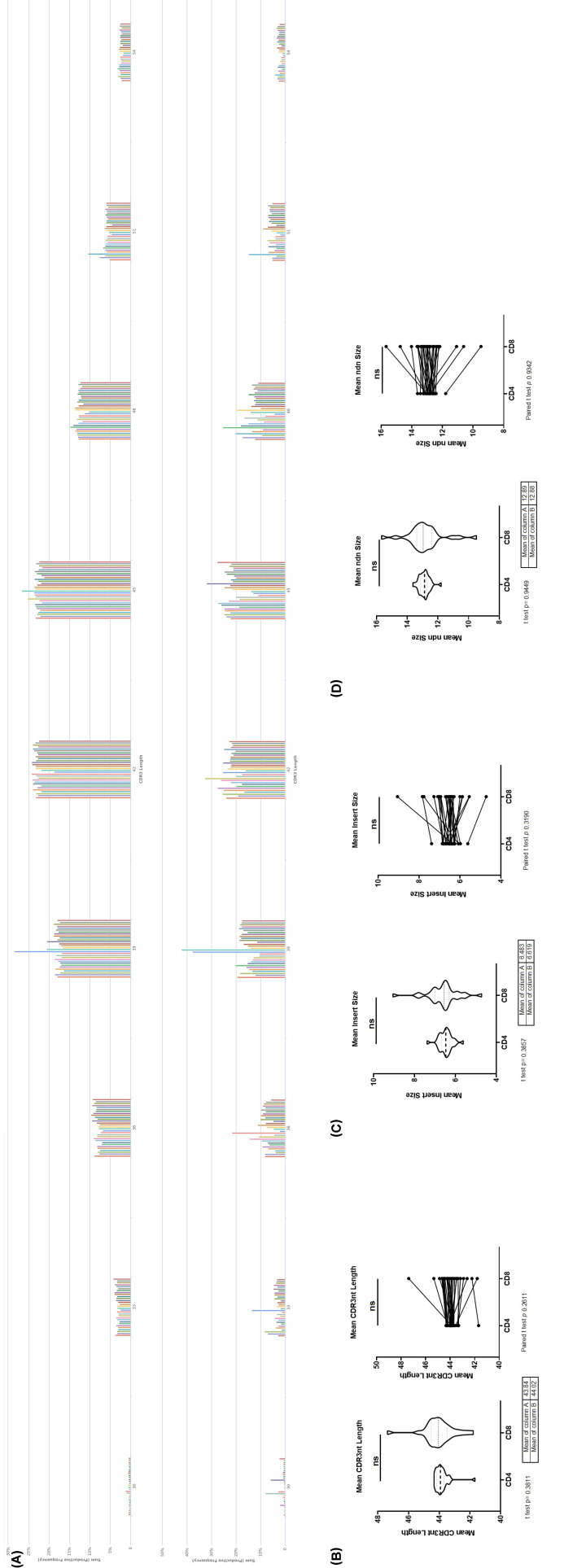
CDR3 length in CD4 and CD8 TCR-β repertoire (**A**) CDR3 length histogram for productive rearrangements frequency. (**B**) The mean length of CDR3 nucleotide sequence, weighted by clonotype frequency. (**C**) The mean number of inserted random nucleotides in CDR3 sequence. (**D**) The mean number of nucleotides that lie between V and J segment sequences in CDR3. (*N* = 30; Left is non-paired *t*-test of CD4 and CD8 TCR-β repertoire as two groups, Right is paired *t-*test of CD4 and CD8 TCR-β repertoire for individual; ns, no significant).

The length of the CDR3 in nucleotides, starting from the first base of the codon for the conserved cysteine in the V gene through the last base of the codon for the conserved residue in the J gene. CDR3 length histogram for productive rearrangements frequency of CD4+ and CD8+TCRβ repertoire from healthy donors are shown in Figure 2A. The mean length of CDR3 nucleotide sequence (Figure 2B), mean number of inserted random nucleotides in CDR3 sequence (Figure 2C), mean number of nucleotides that lied between V and J segment (Figure 2D) are no significant in CD4+ and CD8+TCRβ repertoire from healthy donors

### VDJ usage in CD4 and CD8 TCR-β repertoire

**Figure 3:**
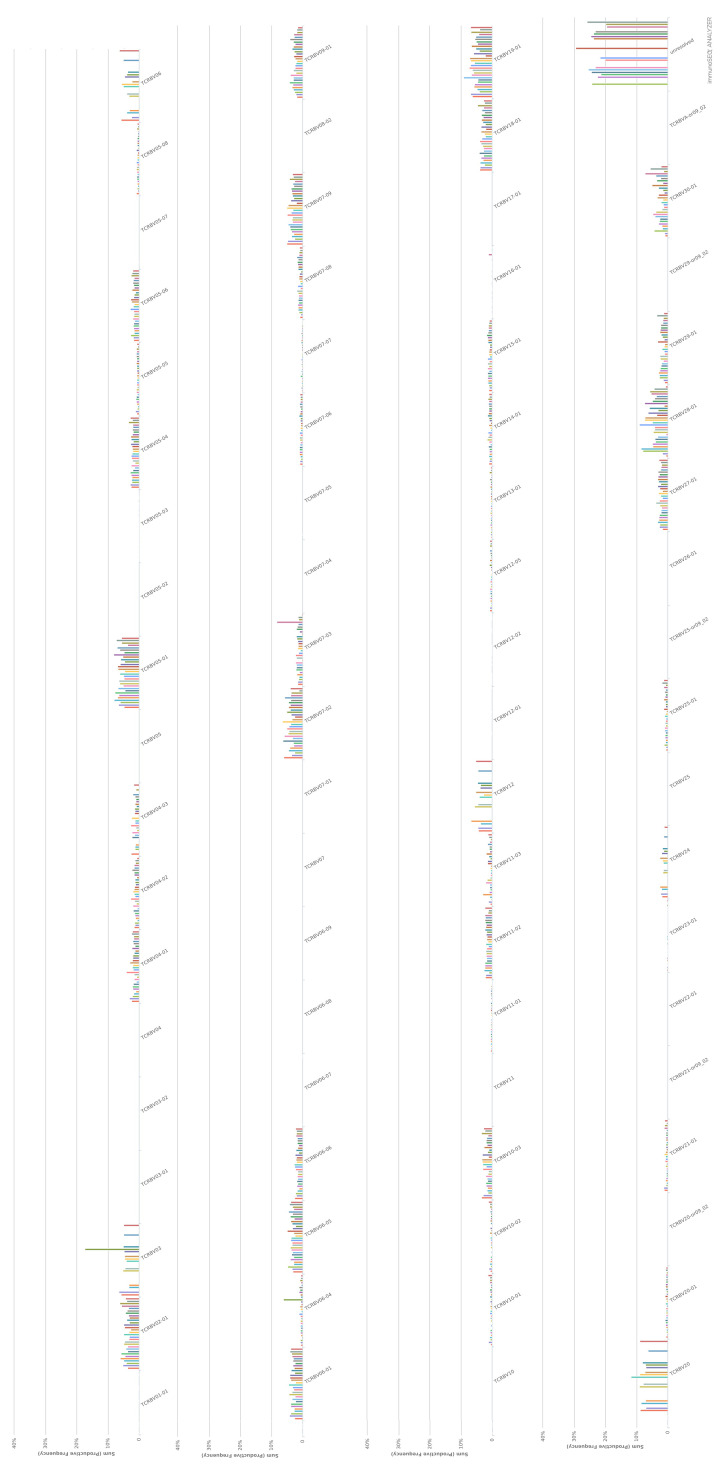
TRBV usage in CD4 TCR-β repertoire

**Figure 4:**
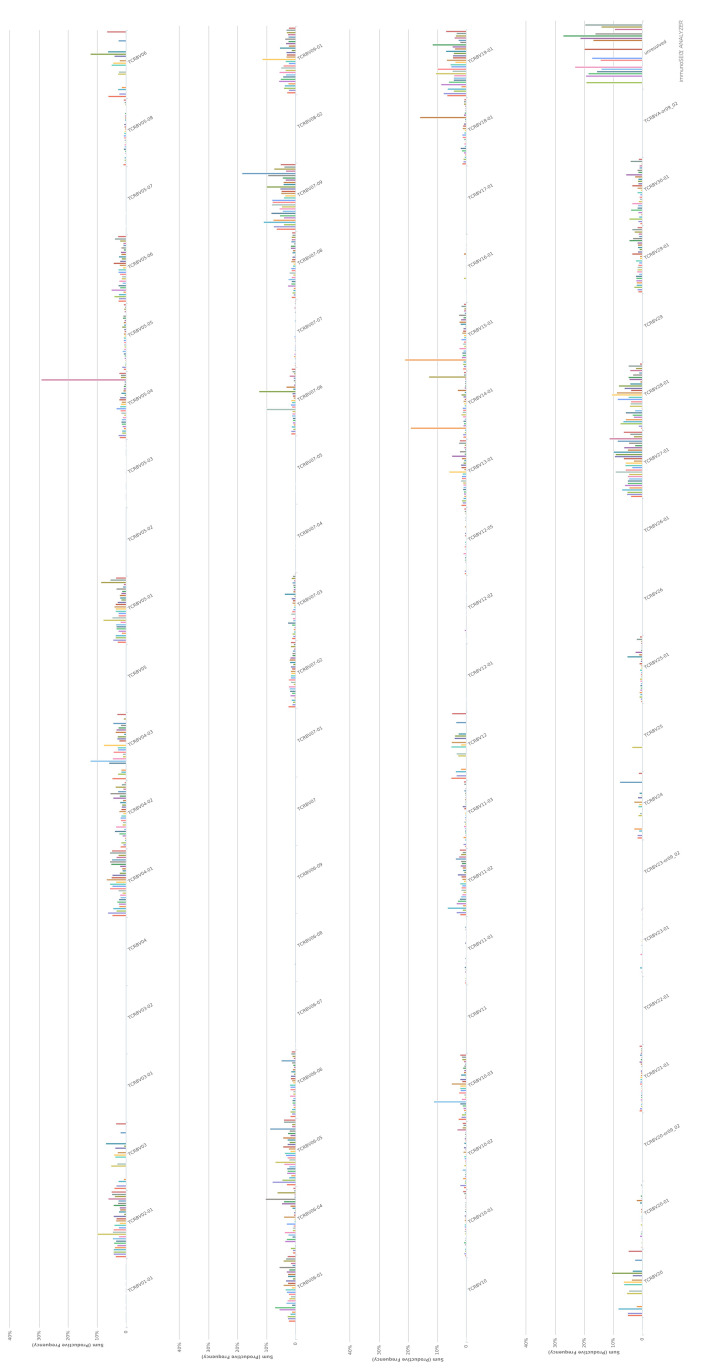
TRBV usage in CD8 TCR-β repertoire

**Figure 5:**
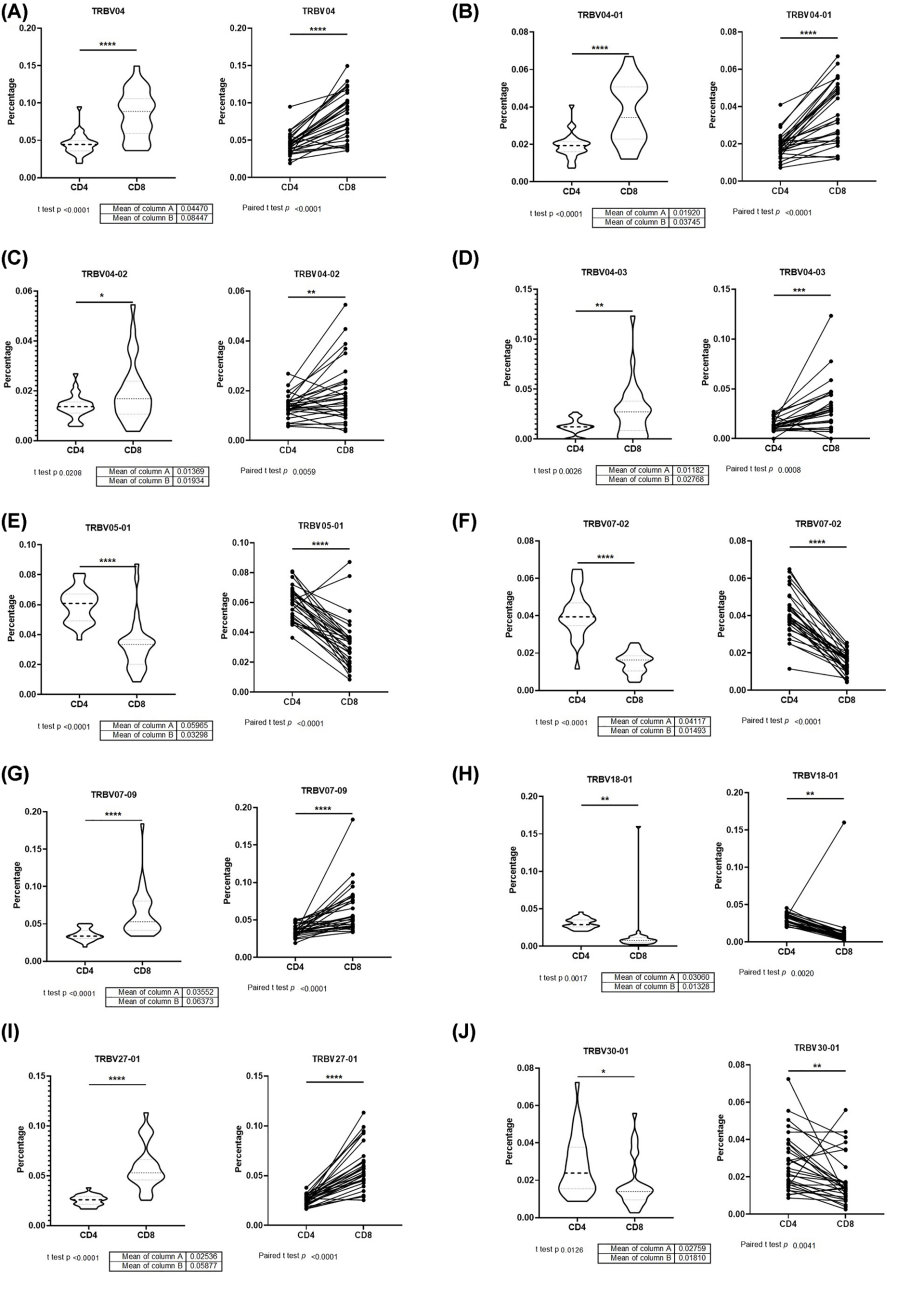
TRBV different usage in CD4 and CD8 TCR-β repertoire (**A**) TRBV04, (**B**) TRBV04-01, (**C**) TRBV04-02, (**D**) TRBV04-03, (**E**) TRBV05-01, (**F**) TRBV07-02, (**G**) TRBV07-09, (**H**) TRBV18-01, (**I**) TRBV27-01, and (**J**) TRBV30-01. (*N* = 30; Left is non-paired *t-*test of CD4 and CD8 TCR-β repertoire as two groups; Right is paired *t-*test of CD4 and CD8 TCR-β repertoire for individual; *P<0.05; **P<0.01; ***P<0.001; ****P<0.0001).

The VDJ usage in CD4 (Figure 3) and CD8 (Figure 4) TCR-β repertoire are shown. The TRBV percentage of TRBV04 (Figure 5A), TRBV04-01 (Figure 5B), TRBV04-02 (Figure 5C), TRBV04-03 (Figure 5D), TRBV07-09 (Figure 5G), and TRBV27-01 (Figure 5I), in CD4+ TCRβ repertoire from healthy donors, are less than CD8+ TCRβ compared with a group or individual. The TRBV percentage of TRBV05-01 (Figure 5E), TRBV07-02 (Figure 5F), TRBV18-01 (Figure 5H), and TRBV30-01 (Figure 5J), in CD4+ TCRβ repertoire from healthy donors, are more than CD8+ TCRβ compared with a group or individual.

### Common top 150 clones VDJ usage

**Figure 6:**
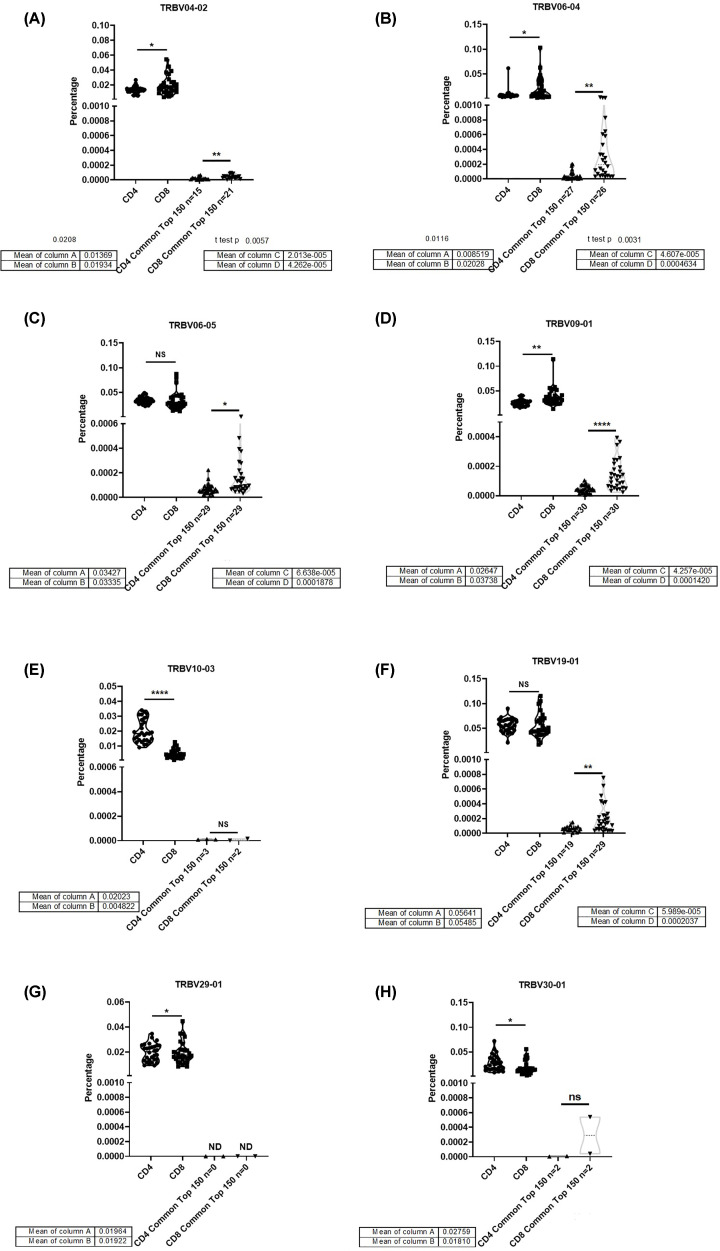
TRBV different usage in CD4 and CD8 common top 150 TCR-β repertoire (**A**) TRBV04-02, (**B**) TRBV06-04, (**C**) TRBV06-05, (**D**) TRBV09-01, (**E**) TRBV10-03, (**F**) TRBV19-01, (**G**) TRBV29-01, and (**H**) TRBV30-01 (ns, no significant; *P<0.05; **P<0.01; ***P<0.001; ****P<0.0001).

Common clones were listed from 30 samples of CD4+ TCRβ repertoire or 30 samples of CD8+ TCRβ repertoire, and sequenced by accumulated frequency. Each sample’s VDJ usage frequency from the top 150 clones was analyzed. The common top 150 clones in TRBV04-02 (Figure 6A), TRBV06-04 (Figure 6B), TRBV06-05 (Figure 6C), and TRBV09-01 (Figure 6D) of CD4+ and CD8+ TCRβ repertoire from healthy donors, which trends similar with bulk TCRβ repertoire. The CD8 common top 150 clones TRBV19-01 (Figure 6F) repertoire have more frequency than CD8+, which has a different trend compared with bulk TCRβ repertoire. The common top 150 clones of TRBV19-01 are found in all samples, which is quite special among other TRBV genes. The common top 150 clone’s repertoire is barely found in TRBV10-03 (Figure 6E), TRBV29-01 (Figure 6G), and TRBV30-01 (Figure 6H), which they have not enough frequency in the bulk repertoire

### Common clone cluster

**Figure 7:**
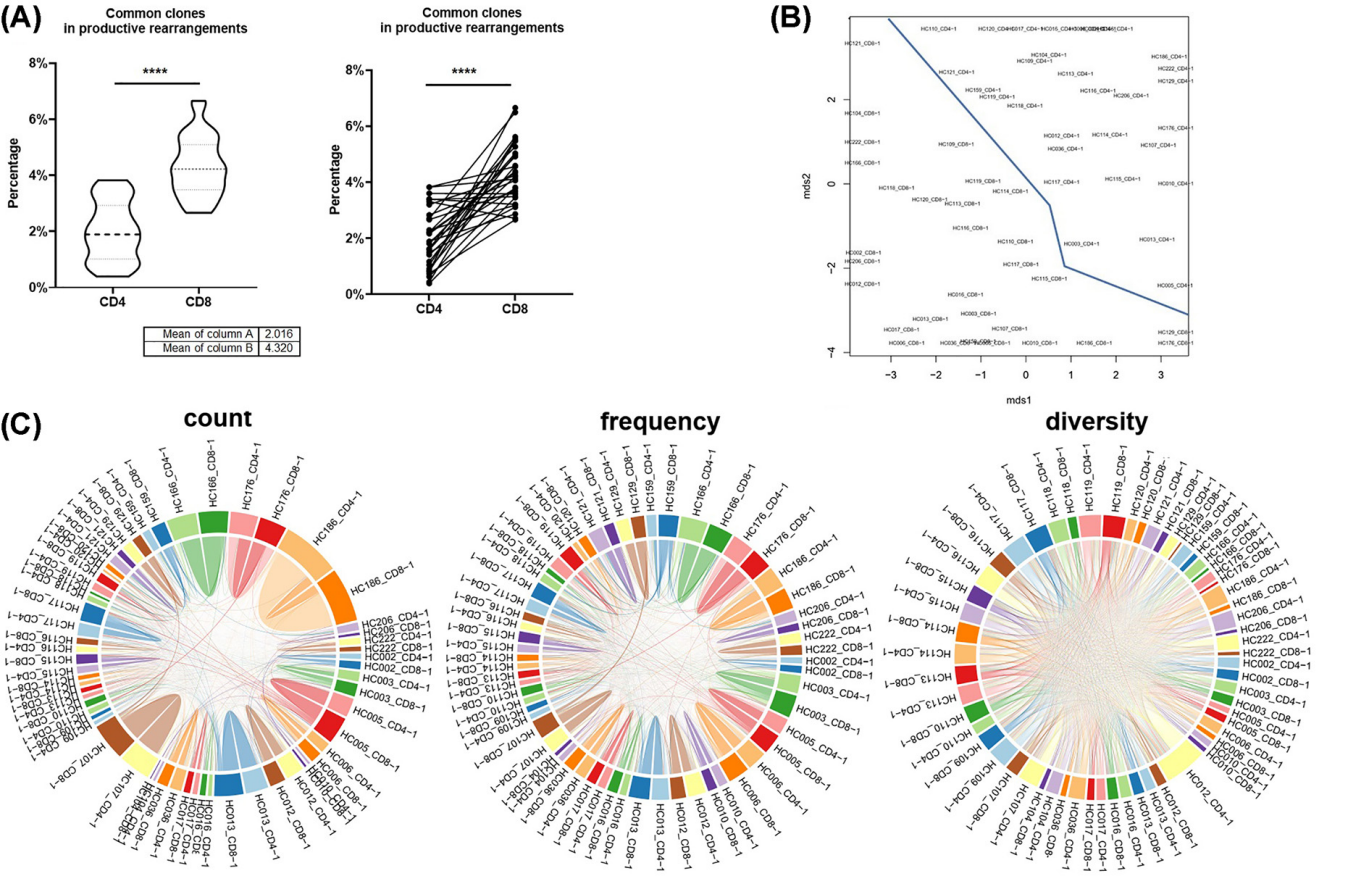
Common Clone Cluster in CD4 and CD8 TCR-β repertoire (**A**) Common clone in productive rearrangements (*N* = 30; Left is non-paired *t-*test of CD4 and CD8 TCR-β repertoire as two groups; Right is paired *t-*test of CD4 and CD8 TCR-β repertoire for individual; ****P<0.0001). (**B**) Multi-dimensional scaling (MDS) for an all-versus-all pairwise overlap of repertoire similarity measures. (**C**) Pairwise overlap circos plot. Count, frequency, and diversity panels correspond to the read count, frequency (both non-symmetric), and the total number of clonotypes that are shared between samples.

**Figure 8:**
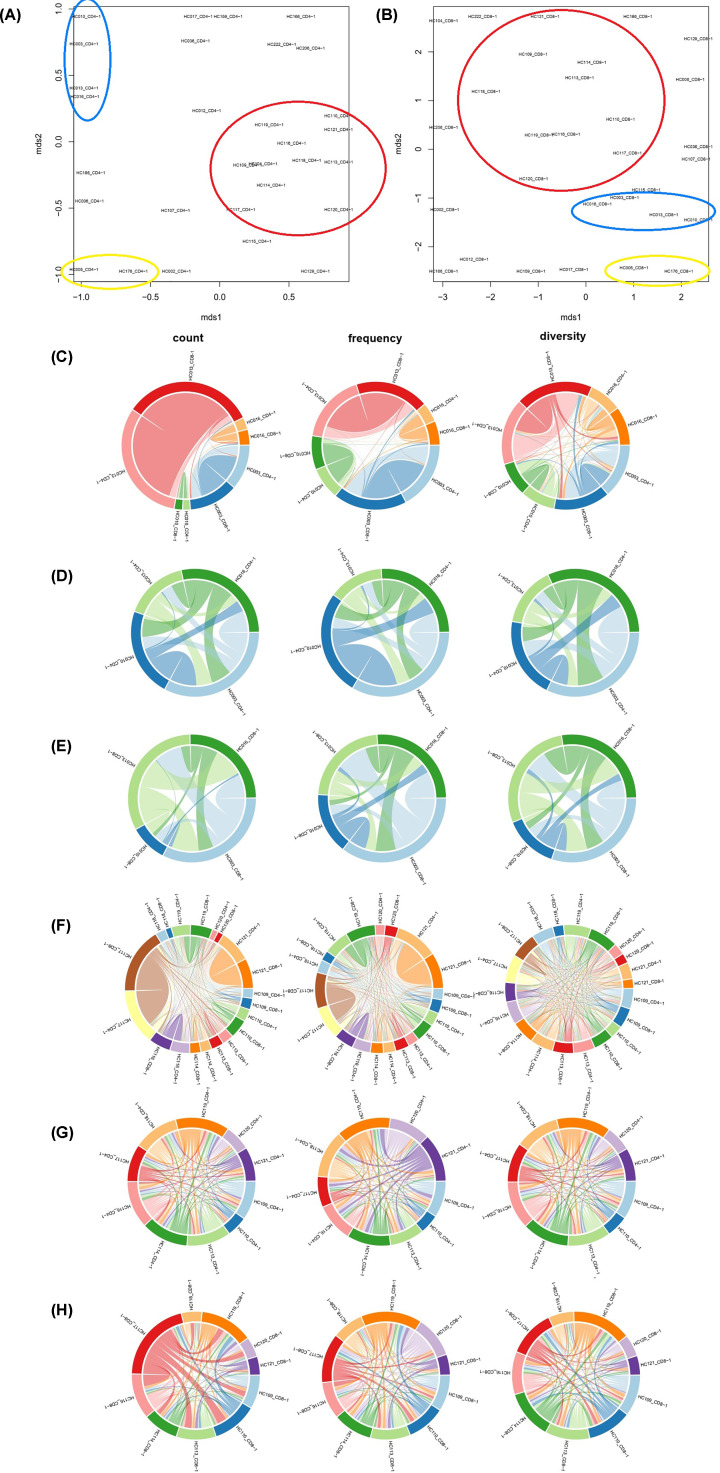
Different individual shared similar TCRβ clones in CD4 and CD8 TCR-β repertoire Multi-dimensional scaling for an all-versus-all pairwise overlap of (**A**) CD4 and (**B**) CD8 TCR-β repertoire similarity measures. Pairwise overlap circos plot. Count, frequency and diversity panels correspond to the read count, frequency and the total number of clonotypes that are shared between samples of Cluster Blue ((**C**) CD4 and CD8, (**D**) CD4, (**E**) CD8 TCR-β repertoire) and Cluster Red ((**F**) CD4 and CD8, (**G**) CD4, (**H**) CD8 TCR-β repertoire).

The common clone frequency in productive rearrangements was analyzed from 30 samples of CD4+ TCRβ repertoire or 30 samples of CD8+ TCRβ repertoire. The common clone frequency of CD4+ TCRβ repertoire from healthy donors is less than CD8+ TCRβ compared with a group or individual (Figure 7A), which showed CD8 TCR clones are more shared and CD4 TCR clones are more unique with a different individual. Multi-dimensional scaling (MDS) for an all-versus-all pairwise overlap of repertoire similarity measures. Pairwise overlap circos plot showed count, frequency, and diversity are shared between samples. The MDS and Pairwise overlap circos plot for 30 samples of CD4+ TCRβ repertoire and 30 samples of CD8+ TCRβ repertoire (Figure 7B), showed that CD4+ and CD8+ TCRβ repertoire could be separated by the line, which means CD4+ TCRβ repertoire and CD8+ TCRβ repertoire are more similar or conserved between different people (Figure 7C). The MDS of 30 samples of CD4+ TCRβ repertoire (Figure 8A) and the MDS of 30 samples of CD8+ TCRβ repertoire (Figure 8B) both showed three groups: Cluster Yellow, Cluster Blue (Figure 8C–E), and Cluster Red (Figure 8C–E), which showed that different individual shared similar TCRβ clones.

## Discussion

In this study, we collected TCRβ repertoire in CD4+ and CD8+ T Cells from 30 health donors from three papers (Supplementary Table S1) [14–16], to find the law of the CD4+ and CD8+ T Cells’ TCRβ clone pattern. The CD4+ TCRβ repertoire has more counts and diversity, less clonality, and mean frequency compared with CD8+ TCRβ (Figure 1A–H). The CD4+ non-coding TCR clone diversity (Figure 1I) has more diversity compared with CD8+ TCRβ, which is the same as the coding clones. The CD4+ and CD8+ CDR3 length (Figure 2A–D) and non-coding TCR frequency (Figure 1J) showed no significance. The non-coding TCR clone is the T-cell preselection repertoire [19]. The result showed that the CD4+ and CD8+ TCRβ repertoire frequencies are the same in the T-cell preselection, but the diversity is made at the beginning, and the frequency changes during T cell maturity and activation (Figure 1A–J).

The VDJ usage in CD4+ and CD8+ TCRβ repertoire showed some preference. TRBV04 (Figure 5A), TRBV04-01 (Figure 5B), TRBV04-02 (Figure 5C), TRBV04-03 (Figure 5D), TRBV07-09 (Figure 5G), and TRBV27-01 (Figure 5I) have more percentage in CD8+ TCRβ repertoire, which TRBV05-01 (Figure 5E), TRBV07-02 (Figure 5F), TRBV18-01 (Figure 5H), and TRBV30-01 (Figure 5J) have more percentage in CD4+ TCRβ repertoire. The VDJ usage of common top clones in CD4+ and CD8+ TCRβ repertoire had different trend compared with bulk TCRβ repertoire (Figure 6E–H), which showed different VDJ usage in CD4+ and CD8+ TCRβ. Each VDJ gene may play a different role in the immune system, which are still a mystery for discovery.

The common clones of CD4+ TCRβ repertoire are less than CD8+ TCRβ (Figure 7A), and bulk CD4+ TCRβ repertoire had more count and diversity (Figure 1A–H), which showed CD8+ TCRβ in a different individual that may because of the same foreign antigen (bacteria or virus). It is interesting that MDS (Figure 7B,C)) result showed that one person's CD8+ TCRβ repertoire is more similar to other people’s, not similar to his/her own CD4+ TCRβ repertoire, may CD8+ and CD4+ TCRβ repertoire have some hidden pattern. The MDS of 30 samples of CD4+ TCRβ repertoire (Figure 8A) and the MDS of 30 samples of CD8+ TCRβ repertoire (Figure 8B) both showed three same groups (Figure 8C–H), so different people have similar TCRβ repertoire may because of the HLA similarity, which may provide a clue that HLA influences the TCR repertoire. Though one person's CD8+ TCRβ repertoire is more similar to other people's than his/her own CD4+ TCRβ repertoire, the same cluster (Figure 8A,B) of CD4+ and CD8+ TCRβ repertoire showed that CD4+ and CD8+ TCR repertoire are connected separately between the bodies.

TCR recognition is vital to defend against infection and tumors [9]. The TCR can recognize peptide antigens (MHC-I, MHC-II, and CD1) and other antigens [20], in which MR1 and HLA-E present metabolites and non-self-lipids. Indicating that T cells have additional roles in immune responses to tissue homeostasis and inflammation [21]. Cancer studies have found that high diversity in the TCR repertoire may be associated with better prognosis [22]. Cancer immunotherapy has recently undergone rapid development for clinical use, such as chimeric antigen receptor (CAR)-T cells and TCR-T cells.

T cell is vital in adaptive immune response, not only in defending against mutation and foreign antigen but also in maintaining immune homeostasis. The recognition and function of T cells rely on TCR, which is diverse and can recognize antigens, but the relationship between the TCR and antigen presentation molecules is still a mystery. Deciphering the secret of TCR diversity and clonality would find a way to uncover the mystery of the immune system.

## Supplementary Material

Supplementary Tables

## Data Availability

Raw TCR-β bulk DNA-seq Data may be accessed using the following link after creating a free account: HC104, HC107, HC109, HC110, HC113, HC114, HC115, HC116, HC117, HC118, HC119, HC120, HC121 are freely accessible through https://clients.adaptivebiotech.com/pub/fu-2021-jci. HC036, HC129, HC159, HC166, HC176, HC186, HC206, HC222 are freely accessible through https://clients.adaptivebiotech.com/pub/savage-2019-ajt. HC002, HC003, HC005, HC006, HC010, HC012, HC013, HC016, HC017 are freely accessible through https://clients.adaptivebiotech.com/pub/gold-2019-cr (Supplementary Table S1). Other information in this study is available on request from the corresponding author. Any materials, data, code and associated protocols (relating to their published research) available to bona fide researcher or reader requests without undue delay or qualifications

## Abbreviations

CAR: chimeric antigen receptor
HLA: human leukocyte antigen
MHC: major histocompatibility complex
TCR: T-cell receptor
